# Assessment of Skeletal Maturity in a Sample of the Saudi Population Using Cervical Vertebrae and Frontal Sinus Index: A Cephalometric Study Using Artificial Intelligence

**DOI:** 10.7759/cureus.41811

**Published:** 2023-07-13

**Authors:** Ahmed A Alfawzan

**Affiliations:** 1 Department of Orthodontics and Pediatric Dentistry, College of Dentistry, Qassim University, Buraydah, SAU

**Keywords:** cervical vertebrae, growth, radiography, sinus index, skeletal maturity

## Abstract

Aim

This study aims to investigate the relationship between the frontal sinus index and different stages of cervical vertebral maturation and to determine whether these measurements can be used as accurate markers for evaluating skeletal maturity.

Materials and methods

The sample size was 120 subjects, with ages that ranged from 8 to 25 years. Standardized pretreatment digital lateral cephalograms were analyzed. Six groups representing different phases of cervical vertebral maturation were created from the entire sample. Each group had 20 subjects and was split into male and female groups. On the same radiograph, the cervical stages were assessed and the frontal sinus index was calculated. Correlations between the frontal sinus index and cervical vertebral maturation were assessed using statistical analysis.

Results

Sinus index values, which assess the ratio of frontal sinus dimensions to cervical stages, were found to be comparable between the two adjacent cervical stages. However, the patterns of sinus index values differed between males and females, suggesting potential variations in sinus development between the sexes.

Conclusion

Frontal sinus height and width were significantly correlated with cervical vertebral maturation stages in both sexes. The frontal sinus index, on the other hand, was not significant.

## Introduction

Analysis of growth is essential to orthodontic treatment. Dynamic variations in body shape and size from birth through adulthood are important aspects of human growth and development. Growth potential evaluation during the preadolescent or adolescent growth spurt plays a key role in determining the orthodontic diagnosis and treatment options. It also has an impact on the stability and effectiveness of orthodontic treatment. Because children of the same chronological age typically have varied levels of skeletal maturation, it is impossible to accurately diagnose a child's growth status based solely on age [1]. There are maturational changes that can be seen from infancy through maturity. It has been used as a natural exhibition of individual skeletal development to observe changes in the size and shape of the cervical vertebrae in growing individuals. By immediately scanning the cervical vertebrae on a lateral cephalogram, an orthodontist can assess the patient's skeletal maturity [2]. The frontal sinus is the last of the sinuses to postnatally develop [3]. It is seen on radiographs at the age of 8 years. By the age of 12 years, it is well expanded [4]. Numerous studies have done extensive research on it, although few have related it to other growth measurements. Research has been done on the enlargement of the frontal sinus from childhood to adulthood and its relationship to the physiological variability of the sinus size. Many researchers have looked for many markers over the years to predict children's skeletal growth. Body weight and height, the menstrual cycle, dental maturity, chronological age, and skeletal maturity are examples of traditional parameters [5].

The role of artificial intelligence (AI) in orthodontics has gained significant attention and it shows promise in improving efficiency and accuracy in orthodontic diagnosis and treatment planning. Cephalometric tracings involve the analysis and measurements of craniofacial structures using standardized X-ray images, known as cephalograms. AI algorithms can be trained to automatically detect anatomical landmarks on cephalometric images. These landmarks are crucial for accurate cephalometric measurements and treatment planning. By using deep learning techniques, AI models can learn to identify landmarks with high accuracy and consistency, reducing the manual effort required by orthodontists. AI can automate the process of measuring various cephalometric parameters. Traditional manual tracing and measurement methods can be time-consuming and subjective. AI algorithms can be trained to perform these measurements accurately and efficiently, leading to standardized and reproducible results. This can improve the consistency and reliability of cephalometric analyses. AI can assist orthodontists in treatment planning by providing automated analysis and predictions. By leveraging machine learning algorithms, AI models can analyze cephalometric data and provide insights into treatment outcomes, growth patterns, and potential treatment options. This can help orthodontists make more informed decisions and optimize treatment plans for individual patients. 

Hand-wrist bone ossification is considered reliable, with the exception of additional radiation exposure. Lamparski linked the use of cervical vertebral maturation to skeletal development to avoid this additional radiation exposure [6]. Similarly, Ruf and Pancherz examined and established the link between frontal sinus expansion and somatic maturity [7]. The aim of this research was to correlate the frontal sinus index and the cervical vertebral maturation to the skeletal maturation using cephalometric radiographs.

## Materials and methods

The pretreatment lateral cephalograms of patients have been obtained from the Department of Orthodontics, Eastern Riyadh Specialist Dental Centre, Riyadh, Kingdom of Saudi Arabia (KSA). The study was approved by the Dental Ethics Committee of Qassim University (approval number: DRC/003FA/23). Good-quality, standardized pretreatment digital lateral cephalograms of the individuals with no history of orthodontic treatment or surgical treatment were included. Patients having a history of sinus-related pathologies, craniofacial deformities, trauma, or surgery involving the frontal sinus or cervical vertebrae, as well as patients with systemic diseases influencing growth and development, were barred from participating.

**Figure 1:**
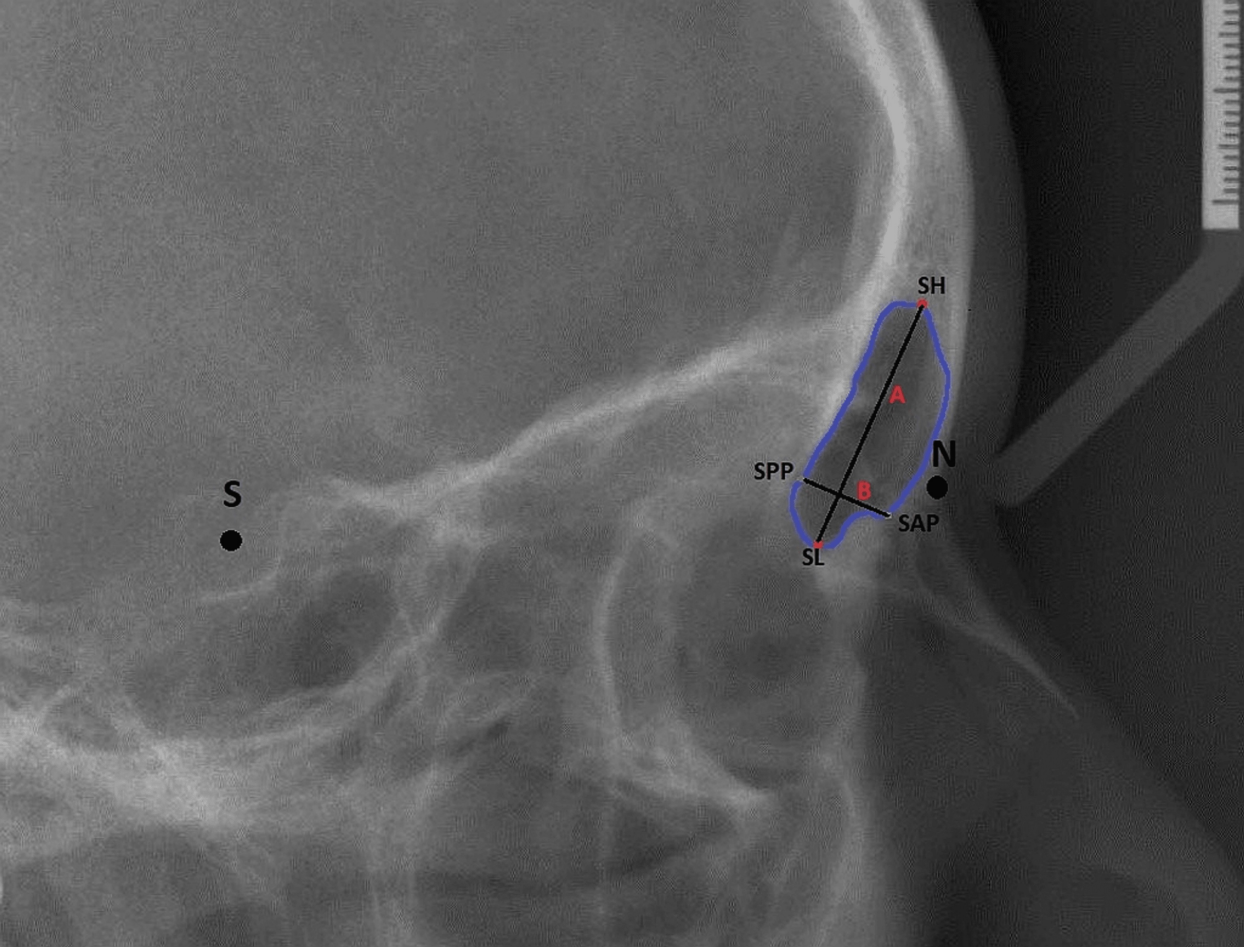
Tracing image from WEBCEPH software Source: Digital image of the lateral cephalogram of the patient included in the study using the artificial intelligence-driven WEBCEPH software.

The sample size consisted of 120 subjects aged between 8 and 21 years. Digital images of the lateral cephalograms were analyzed and measured by one examiner using AI-driven WEBCEPH software (AssembleCircle Corp., Gyeonggi-do, Korea), where WEBCEPH is an FDA 510k-cleared and Korea Food & Drug Administration (KFDA)-approved AI-driven online orthodontic diagnostic software for dentists. WEBCEPH™ is a digital, AI-based, two-dimensional online platform that offers automatic cephalometric tracing, analysis, visual treatment simulation, automatic superimposition, image archiving, and a photo gallery with the benefit of manual landmark editing and automatic measurement calculation (Figure 1).

**Figure 2:**
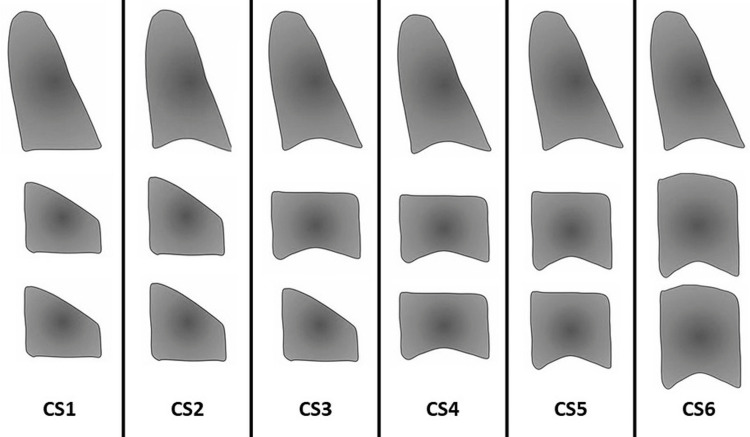
Cervical vertebral maturation stages CS 1: All cervical vertebrae have flat inferior borders and tapering superior borders from posterior to anterior; CS 2: The second vertebra's inferior border concavely changes, and the bodies' anterior vertical height increases; the inferior border of the third vertebrae develops a concavity in CS 3; CS 4: All cervical vertebrae have rectangular bodies, and the inferior border of the fourth vertebrae is developing a concavity; all cervical vertebrae's lower border bodies have clearly defined concavities, are approximately square, and have smaller intervals between them in CS 5; CS 6: The vertebral bodies are now taller than they are wide, and all concavities have deepened. Adapted from Ref. [8].

The total sample was divided into six groups according to Baccetti et al.'s [8] cervical vertebral maturation stages (Figure 2, Table 1).

**Table 1:** Baccetti et al.'s cervical vertebral maturation stages

Stage	Description
Stage 1	Minimal development of cervical vertebrae, with no signs of growth or maturation
Stage 2	Early signs of growth and maturation, characterized by the appearance of concavities on the lower border of the cervical vertebrae
Stage 3	Continued growth and maturation, with increased concavities and the beginning of the formation of the vertebral body
Stage 4	Further growth and maturation, with increased formation of the vertebral body and additional changes in shape
Stage 5	Advanced growth and maturation, characterized by completion of the vertebral body formation and the presence of a distinct ossification center
Stage 6	Completion of growth and maturation, with the fusion of the ossification centers and the cessation of further changes

**Figure 3:**
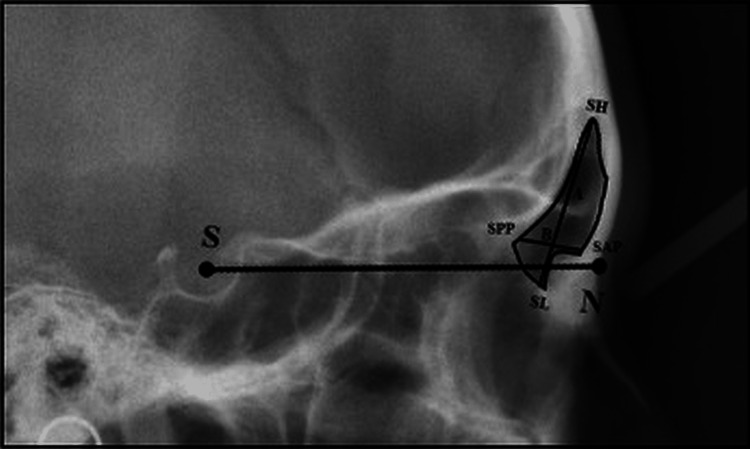
Assessment of frontal sinus morphology SH, the highest point on the frontal sinus; SL, the lowest point on the frontal sinus; A, the line joining SH and SL denoting maximum frontal sinus height; SPP, the posterior point on the frontal sinus; SAP, the anterior point on the frontal sinus; B, the line joining SPP and SAP denoting the maximum frontal sinus width perpendicular to line A; S, the anatomic center of sella turcica; N, the deepest point in the midline at the frontonasal suture. Source: The patient's cephalograph traced traditionally.

Each group consisted of 20 subjects and was further subdivided into males and females. Assessment of frontal sinus morphology on lateral cephalograms was done using the method of Ertuk [5] (Figure 3).

The ratio between height and width is calculated and considered the sinus index, i.e., sinus index = sinus height/sinus width. A descriptive analysis of all the study parameters was done using the mean and SD. The Kruskal-Wallis test was used for the comparison of sinus height, width, and index at different stage groups. The Mann-Whitney test was used for the comparison of sinus height, sinus width, and sinus index between adjacent cervical stages. The level of significance was set at p<0.005.

## Results

Both the width and height of the frontal sinus showed statistically significant increases in both genders. Both the height and width of the frontal sinus were found to be higher in males than in females (Table 2).

**Table 2:** Comparison of sinus height, width, and index at different stage groups

Cervical stage (CS)	Gender	Group 1	Group 2	Group 3	Group 4	Group 5	Group 6	P-Value
Frontal sinus height	Male	19.24 ± 0.58	24.57 ± 0.44	26.45 ± 0.43	32.03 ± 0.58	34.45 ± 0.72	37.43 ± 0.58	<0.001
	Female	18.96 ± 0.48	23.51 ± 0.85	25.4 ± 0.57	28.01 ± 0.81	29.98 ± 0.33	31.73 ± 1.1	<0.001
Frontal sinus width	Male	7.43 ± 0.9	9.21 ± 0.27	10.01 ± 0.45	11.15 ± 0.4	12.93 ± 0.47	15.33 ± 0.48	<0.001
	Female	6.71 ± 0.43	7.54 ± 0.39	8.61 ± 0.45	9.15 ± 0.45	10.88 ± 0.27	11.82 ± 0.85	<0.001
Frontal sinus index	Male	2.62 ± 0.35	2.66 ± 0.1	2.64 ± 0.12	2.87 ± 0.11	2.66 ± 0.12	2.43 ± 0.06	<0.001
	Female	2.83 ± 0.2	3.12 ± 0.16	2.95 ± 0.15	3.07 ± 0.18	2.76 ± 0.08	2.69 ± 0.23	<0.001

When the adjacent cervical vertebral maturation stage groups' frontal sinus height and width were compared, sinus height was statistically significant in both males and females, and sinus width was statistically significant in all groups of males, but in females, it was statistically significant only in group 1 vs. group 2, group 2 vs. group 3, and group 4 vs. group 5 (Table 3).

**Table 3:** Comparison of sinus height, width, and index between the adjacent groups

Variable	Gender	Group 1 vs Group 2 (p-value)	Group 2 vs Group 3 (p-value)	Group 3 vs Group 4 (p-value)	Group 4 vs Group 5 (p-value)	Group 5 vs Group 6 (p-value)
Frontal sinus height	Male	< 0.001	< 0.001	< 0.001	< 0.001	< 0.001
	Female	< 0.001	0.001	< 0.001	< 0.001	< 0.001
Frontal sinus width	Male	0.001	< 0.001	< 0.001	< 0.001	< 0.001
	Female	0.001	< 0.001	0.009	< 0.001	0.019
Frontal sinus index	Male	0.677	0.910	0.001	0.002	< 0.001
	Female	0.005	0.104	0.256	0.001	0.273

The frontal sinus index showed statistical significance between the groups in both males and females. It increased from group 1 to group 2, decreased from group 2 to group 3, and again increased from group 3 to group 4, followed by a decreased pattern from group 4 to group 5 and group 5 to group 6 in both genders. In comparison among the sexes between the adjacent cervical vertebral maturational groups, the results were statistically significant only for group 3 vs. group 4, group 4 vs. group 5, group 5 vs. group 6 in males, and group 1 vs. group 2, group 4 vs. group 5 in females.

## Discussion

It is essential to understand the process and growth potential of the craniofacial region in orthodontic treatment planning. The growth spurt stage during or before adolescence has a significant impact on both orthodontic planning and the retention of results after orthodontic treatment [9]. Observation of changes in the size and shape of the hand and elbow bones on radiographs can be used to identify bone growth, bone formation, and its early manifestations of maturation [10]. Assessment of bone ossification using a hand-wrist radiograph is traditionally considered a reliable indicator of maturation [11, 12]. Evidence points to a complex relationship between genetics, environmental variables, and frontal sinus size [13]. Steiner defined the frontal sinus as the anterior ethmoid air chamber attached to the frontal bone [14]. Since then, many studies have investigated cranial air chamber anatomy and factors predictive of bone growth for medical or surgical purposes [15-17].

Variations in the degree of pneumatization frontal sinus morphology create a remarkable difference in frontal sinus shape, volume, and symmetry. Ruf and Pancherz explain how the frontal sinus develops, how it interacts with other developmental elements, and how it can be used to predict the onset of puberty [7]. They found that by characterizing the development of the central sinuses on lateral head radiographs, the amount of bone and somatic maturation could be predicted with great accuracy. They revealed an association between the development of the frontal sinuses and other developments not found in people of European descent.

In this study, the height of the frontal sinus was gradually increased at the level of the cervical stages and followed a linear pattern in both sexes. Men have larger sinuses than women. Findings for frontal sinus height were similar to those of Mahmood et al., in which frontal sinus height showed a linear pattern of increase [1]. From cervical stage 1 to stage 6, the frontal sinus width also follows a linear pattern of growth. Men have wider sinuses than women. These results agree with those of Pancherz and Ruf. Although they only included males in their sample, they found that the frontal sinus width might be used as a measure of maturity [7].

In this study, when the frontal sinus width on adjacent cervical vertebral maturation was compared, it was statistically significant in all-male groups, while it was statistically significant only in women in GR1 versus GR2, GR2 versus GR3, and GR4 versus GR5. In a study of girls of Japanese descent, Valverde et al. showed that frontal sinus enlargement is associated with increased pressure during puberty [16]. They advocate using changes in frontal sinus morphology as a reliable indicator of growth when assessing a child's development. This study is based on facts. Magnification is a critical element of the cephalometric radiograph, so the ratio is always more reliable than the actual measurement alone [9]. In this study, the frontal sinuses’ height and width increased linearly. In both genders, the frontal sinus index increased from GR1 to GR2, then fell from GR2 to GR3, rose from GR3 to GR4, then decreased from GR4 to GR5, and from GR5 to GRP. When the frontal sinus indices of adjacent groups were compared, the results were statistically significant only between GR3 and GR4, GR4 and GR5, GR5, and GR6 in men, and between GR1 and GR2, GR4 and GR5 in women. There is no specific pattern.

The size of the frontal sinuses grows linearly with age. Although the height and width of the frontal sinus correlated among cervical stages, Mahmood et al. found that the cervical measurement was unable to disclose unique patterns of various cervical stages, and the frontal sinus index failed [1]. Patil and Revankar evaluated the association between the frontal sinus index and the ossification of the middle phalanx of the third finger and found no significant correlation [15]. They found that the frontal sinus index cannot be used as a maturity indicator. The sinus index in this study correlated with the cervical vertebral maturation stage, even if it did not follow a precise pattern.

Posteroanterior and lateral cephalograms were used by Tehranchi et al. to determine the size of the frontal sinus [18]. The link between the size of the frontal sinus and the cephalometric indices was assessed. The findings revealed a relationship between several adult cephalometric measurements and frontal sinus size. Based on these findings, the authors suggest that in young adults, as the frontal sinuses have reached their maximum size, a larger frontal sinus may be linked to future vertical growth. The cervical vertebral maturation (CVM) technique is now the most widely used approach for evaluating children's growth using a lateral cephalogram. The CVM method has advantages in evaluating mandibular growth stages as it can be verified on lateral cephalometric radiographs that are routinely taken for orthodontic diagnosis. The CVM method is closely related to the hand-wrist method and can be utilized in place of the hand-wrist method for evaluating skeletal maturity [19]. However, there are some limitations: for example, cervical vertebral bodies 3 and 4 are trapezoidal, horizontal rectangles, squares, or vertical rectangles [20]. The craniofacial complex includes the stomatognathic system, and the two are closely related. The more we are aware of this relationship, the better we will be able to comprehend and apply orthodontic principles [21].

Rossouw et al. concluded that class III patients with larger sinuses might need orthognathic surgery in addition to future orthodontic appliances [22]. Numerous studies that examined the association between frontal sinus growth and human height found that the frontal sinus may serve as a sign of maturity [9]. The results of this study and other studies indicate that the frontal sinus height and width increase proportionally, with no change in the frontal sinus index values. For a valid evaluation of patient development, a longitudinal study design is necessary. In this cross-sectional investigation, only the frontal sinuses' vertical and sagittal dimensions could be measured on lateral cephalograms. A frontal sinus volume study is recommended to assess their validity as a tool for evaluating various growth stages.

## Conclusions

In conclusion, the research attempted to investigate the relationship between the frontal sinus index and various stages of cervical vertebral maturation to establish whether these measurements can serve as reliable markers for assessing skeletal maturity in patients. The results showed a significant correlation between these parameters, highlighting the potential for using these measurements in clinical practice. However, further research is necessary to validate these findings and determine their usefulness in a broader population.

## References

[1] Mahmood HT, Shaikh A, Fida M (2016). Association between frontal sinus morphology and cervical vertebral maturation for the assessment of skeletal maturity. Am J Orthod Dentofacial Orthop.

[2] Hassel B, Farman AG (1995). Skeletal maturation evaluation using cervical vertebrae. Am J Orthod Dentofacial Orthop.

[3] Lee S, Fernandez J, Mirjalili SA, Kirkpatrick J (2022). Pediatric paranasal sinuses-development, growth, pathology, & functional endoscopic sinus surgery. Clin Anat.

[4] Barghouth G, Prior JO, Lepori D, Duvoisin B, Schnyder P, Gudinchet F (2002). Paranasal sinuses in children: size evaluation of maxillary, sphenoid, and frontal sinuses by magnetic resonance imaging and proposal of volume index percentile curves. Eur Radiol.

[5] Alijani S, Farhadian N, Alafchi B, Najafi M (2020). Relationship of frontal sinus size and maturation of cervical vertebrae for assessment of skeletal maturity. Front Dent.

[6] Lamparski DG (1975). Skeletal age assessment utilizing cervical vertebrae. Am J Orthod.

[7] Ruf S, Pancherz H (1996). Development of the frontal sinus in relation to somatic and skeletal maturity: a cephalometric roentgenographic study at puberty. Eur J Orthod.

[8] Baccetti T, Franchi L, McNamara JA (2005). The cervical vertebral maturation (CVM) method for the assessment of optimal treatment timing in dentofacial orthopedics. Semin Orthod. Sep.

[9] Tehranchi A, Motamedian SR, Saedi S, Kabiri S, Shidfar S (2017). Correlation between frontal sinus dimensions and cephalometric indices: A cross-sectional study. Eur J Dent.

[10] Mohammed RB, Reddy MA, Jain M, Singh JR, Sanghvi P, Thetay AA (2014). Digital radiographic evaluation of hand-wrist bone maturation and prediction of age in South Indian adolescents. Hand (NY).

[11] Bowden BD (1976). Epiphysial changes in the hand/wrist area as indicators of adolescent stage. Aust Orthod J.

[12] Houston WJ, Miller JC, Tanner JM (1979). Prediction of the timing of the adolescent growth spurt from ossification events in hand-wrist films. Br J Orthod.

[13] Chaiyasate S, Baron I, Clement P (2007). Analysis of paranasal sinus development and anatomical variations: a CT genetic study in twins. Clin Otolaryngol.

[14] Karakas S, Kavakli A (2005). Morphometric examination of the paranasal sinuses and mastoid air cells using computed tomography. Ann Saudi Med.

[15] Patil AA, Revankar AV (2013). Reliability of the frontal sinus index as a maturity indicator. Indian J Dent Res.

[16] Guevara Y, Watanabe N, Yamaki M, Saito I (2013). The frontal sinus enlargement as an indicator of growth maturity in class III patients - a pilot study. Int J Med Sci Public Health.

[17] Gagliardi A, Winning T, Kaidonis J, Hughes T, Townsend GC (2004). Association of frontal sinus development with somatic and skeletal maturation in Aboriginal Australians: a longitudinal study. Homo.

[18] Tehranchi A, Saedi S, Motamedian S, Rohani K (2015). Radiographic evaluation of frontal sinus dimensions and anatomic variations. Br J Med Med Res.

[19] Wong RW, Alkhal HA, Rabie AB (2009). Use of cervical vertebral maturation to determine skeletal age. Am J Orthod Dentofacial Orthop.

[20] Nestman TS, Marshall SD, Qian F, Holton N, Franciscus RG, Southard TE (2011). Cervical vertebrae maturation method morphologic criteria: poor reproducibility. Am J Orthod Dentofacial Orthop.

[21] Said OT, Rossouw PE, Fishman LS, Feng C (2017). Relationship between anterior occlusion and frontal sinus size. Angle Orthod.

[22] Rossouw PE, Lombard CJ, Harris AM (1991). The frontal sinus and mandibular growth prediction. Am J Orthod Dentofacial Ortho.

