# Correction: Assessment of Skeletal Maturity in a Sample of the Saudi Population Using Cervical Vertebrae and Frontal Sinus Index: A Cephalometric Study Using Artificial Intelligence

**DOI:** 10.7759/cureus.c413

**Published:** 2026-03-17

**Authors:** Ahmed A Alfawzan

**Affiliations:** 1 Department of Orthodontics and Pediatric Dentistry, College of Dentistry, Qassim University, Buraydah, SAU

This article has been corrected to change the author’s affiliation from Department of Preventive Dentistry, Qassim University, Ar Rass, SAU to Department of Orthodontics and Pediatric Dentistry, College of Dentistry, Qassim University, Buraydah, SAU.

